# Effect of Clinical Inertia on Diabetes Complications among Individuals with Type 2 Diabetes: A Retrospective Cohort Study

**DOI:** 10.3390/medicina58010063

**Published:** 2021-12-31

**Authors:** Piranee Kaewbut, Natapong Kosachunhanun, Arintaya Phrommintikul, Dujrudee Chinwong, John J Hall, Surarong Chinwong

**Affiliations:** 1 PhD’s Degree Program in Pharmacy, Faculty of Pharmacy, Chiang Mai University, Chiang Mai 50200, Thailand; piranee.ka@up.ac.th; 2 Department of Pharmaceutical Care, School of Pharmaceutical Sciences, University of Phayao, Phayao 56000, Thailand; 3 Department of Internal Medicine, Faculty of Medicine, Chiang Mai University, Chiang Mai 50200, Thailand; natapong.k@cmu.ac.th (N.K.); arintayap@yahoo.com (A.P.); 4 Department of Pharmaceutical Care, Faculty of Pharmacy, Chiang Mai University, Chiang Mai 50200, Thailand; dujrudee.c@cmu.ac.th; 5 Center of Excellence for Innovation in Analytical Science and Technology for Biodiversity-Based Economic and Society (I-ANALY-S-T_B.BES-CMU), Chiang Mai University, Chiang Mai 50200, Thailand; 6 School of Public Health and Community Medicine, University of New South Wales, Sydney, NSW 2052, Australia; john.hall@unsw.edu.au

**Keywords:** clinical inertia, treatment intensification, type 2 diabetes, macrovascular complications, microvascular complications, diabetic nephropathy

## Abstract

*Background and Objectives*: Clinical inertia is a key obstacle that leads to suboptimal care in patients with type 2 diabetes mellitus (T2DM). It can occur at any stage of T2DM treatment. However, the effect of clinical inertia on diabetes complications has not been studied sufficiently. This study aimed to evaluate the effect of clinical inertia on the risk of diabetes complications among patients with T2DM. *Materials and Methods*: A retrospective cohort study was conducted at a tertiary teaching hospital in Thailand between 2011 and 2017. Outpatients with T2DM, aged 40–65 years, presenting an HbA1c greater than 7% were included in this study. Clinical inertia was identified when patients did not get treatment intensification at the index date and a subsequent prescription. The association between clinical inertia and diabetes complications, including a composite of macrovascular complications and a composite of microvascular complications, was determined using a Cox proportional hazard model. Propensity score methods were applied, to control confounding by indication. *Results*: Of 686 patients with T2DM, 165 (24.0%) experienced clinical inertia. Baseline low-density lipoprotein cholesterol, blood pressure, body mass index, the estimated glomerular filtration rate, and medication between the two groups did not differ significantly. Our study found that clinical inertia was associated with a significantly increased risk of diabetic nephropathy (adjusted HR 1.51, 95% CI 1.01–2.27). The results remained the same as when using propensity score methods. According to the post hoc analysis, lowering the HbA1c levels by 1% results in a significant decrease in the rate of diabetic complications (adjusted HR 0.92, 95% CI 0.86–0.99), the composite of microvascular complications (adjusted HR 0.91, 95% CI 0.84–0.98) and diabetic nephropathy (adjusted HR 0.89, 95% CI 0.80–0.98). *Conclusions*: Our results demonstrated a significant effect of clinical inertia on diabetic nephropathy. Patients with an HbA1c level over the target range should have their medication intensified to reduce the risk of diabetic nephropathy.

## 1. Introduction

Diabetes is a common chronic, complex, and progressive disease that affected 463 million people worldwide in 2019 [1]. Type 2 diabetes mellitus (T2DM), accounting for around 90% of diabetes cases [2], is characterized by high insulin resistance and inadequate insulin production, resulting in high glycemic levels [3,4]. Inadequate glycemic control can lead to macro- and microvascular complications for the individual [5].

Reliable evidence has confirmed that maintaining the glycemic level within recommended targets early in the course of the disease is beneficial in reducing complications [6,7,8,9,10,11]. The American Diabetes Association (ADA) recommends that the glycated hemoglobin A1c (HbA1c) target range for non-pregnant adults with diabetes is <7.0% [12]. The Thai Clinical Practice Guideline recommends that newly diagnosed patients without complications or comorbidities should aim for strict control (HbA1c target < 6.5%), but when the patients present hypoglycemia and weight gain, the HbA1c target should be <7.0% [13]. According to these diabetes guidelines, the recommended first step for the patient is lifestyle modification, exercise, and diet control. If the HbA1c still does not reach the target, the next step is to commence first-line pharmacologic treatment, depending on comorbidities, patient-centered treatment factors, and management needs [12]. If the patient’s glycemic level is not within target after three months, treatment intensification with further antidiabetic drugs is recommended [12,13].

Despite good-quality evidence and recommendations, achieving and maintaining such glycemic control is known to be universally difficult [14]. From 2007 to 2010, data from the National Health and Nutrition Examination Surveys (NHANES) showed that people with T2DM and adequate glycemic control (HbA1c < 7.0%) were reported in only 52.5% of cases [15]. Clinical inertia has been identified as one of the key reasons for failing to achieve the HbA1c target [14,16]. Therapeutic inertia is a synonym for clinical inertia [17]. L. S. Phillips et al. coined the term “clinical inertia” in 2001, coming up with the first definition based on logical reasoning [18]. Okonufa et al. coined the term “therapeutic inertia” in 2006, identifying the same concept [19]. Clinical inertia is defined as the failure to enhance drug therapy when clinically indicated [20]. The extent of clinical inertia in diabetes management ranges from 8.4 to 70%, depending on the research method and the country where the study was performed [21]. In the United States, the prevalence of clinical inertia ranges from 28 to 73% [22]. In Thailand, clinical inertia ranges from 26.2 to 68.4% [23,24,25]. This phenomenon is of increasing concern in diabetes management worldwide [26,27]. A recently published study found that clinical inertia has been associated with the progression of diabetic retinopathy [23]. Further, continued clinical inertia can lead to macrovascular complications and mortality [28,29]. Finally, an additional consequence of clinical inertia is the increased cost on healthcare systems, resulting from the treatment of diabetes complications [30].

In Thailand, a published study investigated the prevalence and associated factors of type 2 diabetes [24]. The study found that 26.2% of patients experienced clinical inertia. The use of insulin, the glycated hemoglobin (HbA1c) level at the index date, the number of antidiabetic drugs used, and treatment by specialists were associated with clinical inertia. The factors associated with clinical inertia were used to predict a propensity score in our study. The effect of clinical inertia on diabetes complications has not been studied adequately. This study aimed to evaluate the effect of clinical inertia on the risk of macrovascular and microvascular complications among patients with T2DM. Clinical inertia can exist at any step throughout the therapy of T2DM [31]. Our study’s classification of clinical inertia will assess clinical inertia covering almost all stages of treatment and matching the real-life situation.

## 2. Materials and Methods

### 2.1. Study Design and Setting

This retrospective cohort study was registered with the Thai Clinical Trial Registry with the identification number: TCTR20180129004 and was approved by the Ethics Committee at the Faculty of Medicine, Chiang Mai University, Thailand (certificate of approval, NONE-2560-05209). It was carried out in accordance with the relevant rules and legislation. Informed consent was exempted or not required due to the policy and the type of research.

Medical records and the computerized hospital database of the Maharaj Nakorn Chiang Mai Hospital, a tertiary teaching hospital in northern Thailand, were used to collect patient data from January 2011 to December 2011, with a follow-up on December 2017.

### 2.2. Identification of Subjects

#### 2.2.1. Inclusion Criteria

In 2011, all patients with T2DM attending the outpatient clinics of Maharaj Nakorn Chiang Mai Hospital who met the following criteria were eligible for enrollment: (1) aged 40 to 65 years with a diagnosis of T2DM; (2) HbA1c ≥ 7.0%; and (3) taking at least one oral antidiabetic drug (OAD).

#### 2.2.2. Exclusion Criteria

The exclusion criteria were as follows: (1) a history of symptomatic hypoglycemia; (2) pregnancy and/or lactation; (3) end-of-life care; (4) insulin as the sole medication; (5) the presence of multiple comorbidities, defined as having a Charlson comorbidity score of at least 3; and (6) poor, inconsistent lifestyle modification and medication adherence.

### 2.3. Definition of Terms

From January to December 2011, the index date was defined as the first HbA1c laboratory test date over the target threshold (HbA1c < 7.0%).

Clinical inertia was identified when patients did not get treatment intensification at the index date and a subsequent prescription.

The lack of clinical inertia was identified in two ways. Firstly, that patients were given treatment intensification at the index date or the subsequent prescription. Secondly, that patients were given delayed treatment intensification at the index date but had a blood sugar test within the target level at the time of the subsequent prescription.

Treatment intensification was defined as increasing an existing OAD dosage, switching from an OAD to an injectable antidiabetic medicine, or adding a new OAD without discontinuing or decreasing the dose of existing OADs.

### 2.4. Study Variables and Measurement

After enrollment, each subject’s baseline characteristics were recorded, including age, sex, duration of diabetes, health insurance plan, smoking and drinking status, HbA1c, the existence of comorbidities and/or diabetes complications, history of cardiovascular disease, history of diabetic nephropathy, history diabetic of retinopathy, body weight, blood pressure, lipid profile, estimated glomerular filtration rate (eGFR), medications, insulin use and the type of physician.

The Charlson comorbidity index (CCI) score was used to evaluate comorbidity. CCI scores predict the ten-year survival rate for a patient who may have a range of comorbid conditions, such as myocardial infarction (MI), liver disease, or renal disease. Each condition is assigned a score of one, two, three, or six depending on the risk level [32].

Participants were classified as having multiple comorbidities when they had a CCI score of at least three.

Poor lifestyle modification or medication adherence was identified when the physician or health care provider noted in the medical record that the patient had not made any lifestyle modifications, demonstrated medication nonadherence, or had missed an appointment.

The main health insurance plans in Thailand include universal health care coverage (UC), the Civil Servant Medical Benefit Scheme (CSMBS), social health insurance (SHI), and self-pay [33].

General practitioners, residents, and specialists were identified as the type of physicians in question. General practitioners were defined as externs and interns. Specialists included Fellows and staff physicians.

The following information, including HbA1c, LDL-C, medications, and diabetes complications, was recorded from the index date until the last follow-up.

HbA1c at the last visit was defined as the HbA1c level at the time of occurrence of diabetes complications or the most recent level. If the patients did not have diabetes complications, HbA1c was defined as the last value of the study period.

### 2.5. Study Outcomes

**Figure 1:**
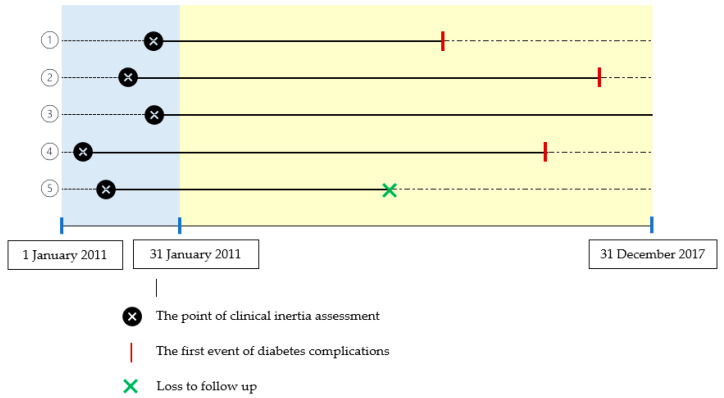
Clinical inertia assessment and diabetes complications. For example, of five patients who were assessed for clinical inertia in 2011 and were then followed up for diabetes complications from 2011 to 2017, three patients had diabetes complications (1, 2, 4); one patient had no diabetes complications until the end date of follow-up (31 December 2017) (3); one patient was lost to follow-up (5).

Patients were followed for at least six years from the date of clinical inertia assessment until the first occurrence of diabetes complications, or until December 2017, whichever occurred first, or until the final entry on the patient’s medical record. The period between the dates of clinical inertia assessment and the first diabetes complications was designated as the time to diabetic complications (Figure 1).

Diabetes complications in the study included a composite of macrovascular and microvascular complications.

The composite of macrovascular complications included the occurrence of myocardial infarction (MI), ischemic stroke, or heart failure (HF).

The composite of microvascular complications comprised the occurrence of diabetic nephropathy (DN) or diabetic retinopathy (DR).

### 2.6. Statistical Analysis

For all statistical studies, Stata Software Version 14 was utilized. Categorical variables were described using frequencies and percentages. Continuous variables were described using the mean ± standard deviation (SD) or median (interquartile range (IQR)). In terms of categorical data, Fisher’s exact test was employed to look for differences across groups. The independent *t*-test or Mann–Whitney U test were employed for continuous data, as appropriate. The hazard ratios (HRs) and 95-percent confidence intervals (95% CIs) were calculated using Cox proportional hazard models.

#### 2.6.1. Propensity Score Methods

Our study employed three propensity score methods to adjust for confounding between the experiencing clinical inertia and no clinical inertia groups: propensity score-matched analysis, covariate adjustment, and inverse probability of treatment weighting (IPTW).

The propensity score method was derived from an unconditional logistic regression model. This analysis was used to reduce bias by equating the two groups based on the following variables: HbA1c, the number of drugs used, insulin, and the type of physician. The variables were based on a previous study looking at factors affecting the clinical inertia in the same setting [24].

Propensity score matching (PSM) between the groups was carried out using a 1:1 ratio. A standardized mean difference (SMD) of <0.1 was used to indicate an adequate covariate balance between groups. Propensity score covariate adjustment and IPTW analysis were also carried out using the same variables to confirm the results of the study. In the IPTW approach, patients in the clinical inertia group were assigned a weight of 1, whereas patients in the no clinical inertia group were assigned a weight of ps/(1−ps), where ps is the propensity score. After weighting, the difference in patient characteristics between the two groups was measured using the standardized difference (STD). A significant difference between groups was defined if an absolute STD was more than 10%. Any variables that remained imbalanced after weighting would be corrected for double robustness.

The multivariable Cox proportional hazard model was also used to assess the effect of clinical inertia on diabetes complications. In all cases, a result that was two-tailed and at a *p*-value < 0.05 was considered statistically significant.

#### 2.6.2. Post Hoc Analysis

Post hoc analysis was used to study the changes of HbA1c level during the last visit of the study, compared with baseline, and the association between the changes of HbA1c level from baseline and diabetes complications.

## 3. Results

### 3.1. Baseline Characteristics

**Figure 2:**
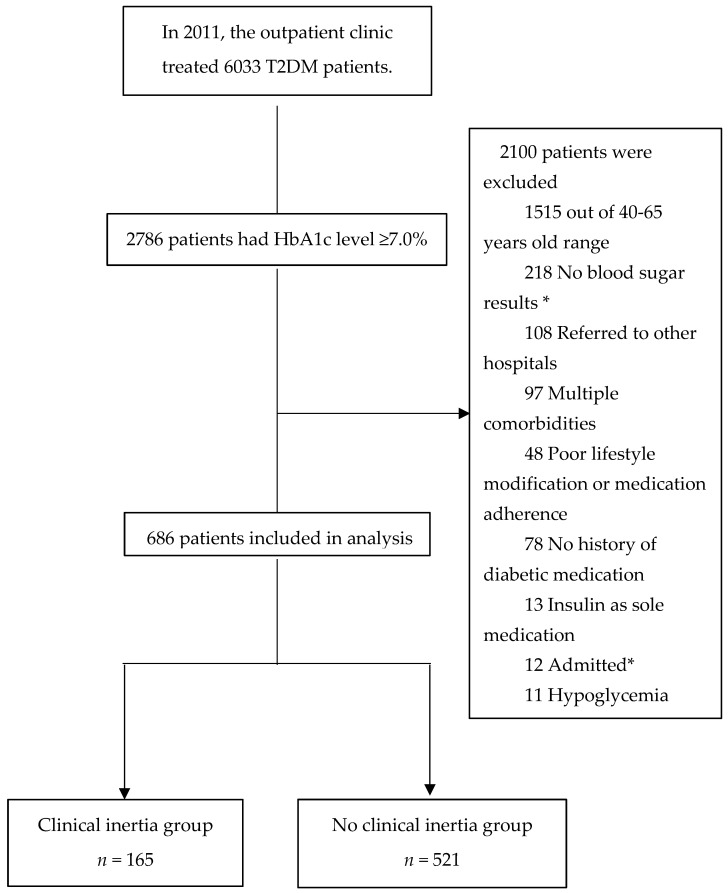
Patient enrollment flow diagram. * No blood sugar results at subsequent visit after the index date. “Admitted” was defined as patients who received inpatient treatment during the assessment of clinical inertia.

A total of 6033 T2DM outpatient medical records were reviewed for enrollment. Of these, 2786 patients had an HbA1c level ≥ 7.0%. After excluding 2100 patients, 686 patients were included in the final analysis (Figure 2).

In the cohort of 686 patients: 43.3% were female, 4.5% were current smokers, and the mean ± SD of age was 53.59 ± 6.04 years. The median (IQR) duration of T2DM was 5 (3–6) years; the mean ± SD of HbA1c at baseline was 8.32 ± 1.29; 14.1% used insulin; 45.3% were covered by the CSMBS; and 44.2% were treated by general practitioners. Of the included patients, 7.7, 10.1 and 7.7% had a history of cardiovascular disease, DN, and DR, respectively.

A comparison between the two groups of clinical inertia and no clinical inertia showed no significant difference in baseline characteristics, except sex, DR, HbA1c at baseline, and HDL-C. The mean HbA1c at baseline was significantly lower among participants who had clinical inertia than among those in the no clinical inertia group (*p* < 0.001). DR was slightly higher in the no clinical inertia group compared with the clinical inertia group (*p*-value = 0.002) as shown in Table 1.

### 3.2. Effect of Clinical Inertia on Diabetes Complications

In this study, 165 patients (24.0%) experienced clinical inertia. During 6.48 years of median follow-up, 67 (40.6%) patients in the clinical inertia group and 211 (40.5%) patients in the no clinical inertia group experienced diabetes complications (Table 2). Multivariable Cox proportional hazard models in the overall cohort analysis showed that clinical inertia significantly increased the risk of DN (adjusted HR 1.51, 95% CI 1.01–2.27).

#### 3.2.1. Macrovascular Complications

The composite of macrovascular complications occurred more frequently in the clinical inertia group (6.7%) than in the no clinical inertia group (5.8%). However, this difference did not reach statistical significance (adjusted HR 1.24; 95% CI (0.60–2.59)). Compared with the no clinical inertia group, clinical inertia was not associated with each component of the composite of macrovascular complications as shown in Table 2.

#### 3.2.2. Microvascular Complications

Although the study found that clinical inertia significantly increased the risk of DN, no significant differences were found between the two groups for the composite of microvascular complications and DR, as shown in Table 2. A total of 259 patients had the composite of microvascular complications: 38.8% in the clinical inertia group, compared with 37.4% in the no clinical inertia group (adjusted HR 1.16; 95% CI (0.86–1.55)). DR was recorded in 40 of 165 patients in the clinical inertia group versus 142 of 521 patients in the no clinical inertia group (adjusted HR 0.95; 95% CI (0.66–1.36)).

### 3.3. Propensity Score Study

The 165 patients in the no clinical inertia group were matched with the 165 patients in the clinical inertia group who had the closest propensity scores. In the matched group, the baseline characteristics of the patients were presented in Table 1. Similar to the overall cohort, patients in the propensity score-matched cohort showed a statistically significant difference regarding DN (adjusted HR 1.72; 95% CI (1.03–2.88)), as shown in Table 2.

Nevertheless, clinical inertia did not increase the risk of other diabetic complications. Sixty-seven patients (40.6%) in the clinical inertia group had diabetes complications compared with 67 patients in the no clinical inertia group (40.6%), corresponding to an adjusted HR of 1.24 (95% CI 0.87–1.77). Patients in the two groups had similar exposure to composite of macrovascular complications (adjusted HR 1.33, 95% CI 0.52–3.44) and a composite of microvascular complications (adjusted HR 1.34, 95% CI 0.93–1.92). The results remained the same after adjusting for propensity score and IPTW (Table A1 and Table A3, available as Appendix A).

### 3.4. Post Hoc Analysis

Our study found that in the clinical inertia group, the differences in HbA1c at the last visit of the study (7.95 ± 1.58), compared with the baseline (8.03 ± 1.04), were not significant (*p*-value = 0.512). In the no clinical inertia group, HbA1c decrements at the last visit (8.06 ± 1.63) of the study from baseline (8.41 ± 1.35) were significant (*p*-value < 0.001) as shown in Table 3.

The difference in mean baseline HbA1c between the clinical inertia group (8.03 ± 1.04) and the no clinical inertia group (8.41 ± 1.35) was significant (*p*-value < 0.001) as shown in Table 1. Conversely, the differences in mean HbA1c at the last visit between the two groups were insignificant. These results are summarized in Table 3.

Moreover, a post hoc analysis was used to study the association between the changes of HbA1c level from baseline and diabetes complications using the Cox proportional hazard model. Our study showed that a decrease in HbA1c by 1% significantly decreased the occurrence rate of diabetes complications (adjusted HR 0.92, 95% CI (0.86–0.99)), composite of microvascular complications (adjusted HR 0.91, 95% CI (0.84–0.98)), and DN (adjusted HR 0.89, 95% CI (0.80–0.98)), as shown in Table 4.

## 4. Discussion

Our real-life situational study investigated the effect of clinical inertia on diabetes complications among patients with T2DM attending a tertiary teaching hospital in northern Thailand. A propensity score method, including matching, covariate adjustment and IPTW, was used to address confounding in this study. Confounding by indication refers to a bias in the connection between a particular treatment and the desired outcome, given the choice of treatment. The physician’s and patient’s evaluations of disease severity, prognosis, and predicted therapeutic impact of the treatment are used to determine the indications for treatment [34]. Our study used three models of propensity score methods to establish a balanced distribution of confounders across treatment groups and minimize clinical factors that impact treatment decisions.

In the multivariable Cox proportional hazard model, this cohort study found that the existence of clinical inertia had a significant effect on DN. The results remained the same as propensity score matching, covariate adjustment, and IPTW.

### 4.1. Macrovascular Complications

Clinical inertia was not significantly associated with a composite of macrovascular complications. Our result was in contrast with Paul et al.’s study [29]. Paul et al., who evaluated the effect of delay in treatment intensification (TI) together with poor glycemic control on the risk of cardiovascular events (CVE) among patients with T2DM, found that patients presenting HbA1c ≥ 7.0% with treatment intensification after 1 year had a significantly increased risk of MI, HF, stroke and any CVE compared with patients presenting HbA1c < 7.0%, with treatment intensification within 1 year. We acknowledge that a possible explanation for this result may be that the results were an effect of delayed TI in conjunction with uncontrolled blood sugar. Thus, the effect may be caused by uncontrolled blood sugar. According to meta-analysis studies, intensive glycemic control reduced the risk of MI, coronary heart disease, and major CVE by 15 to 17%, 15%, and 9%, respectively [35,36]. Moreover, HbA1c levels had significant long-term implications for the likelihood of diabetic complications [37]. Failure to achieve the HbA1c target can result in diabetes complications [38].

A study found that T2DM patients with a history of microvascular complications are at a very high risk of cardiovascular disease [39]. In our study, the overall cohort at baseline, the no clinical inertia group had a history of DR more significantly than the clinical inertia group. This may explain why myocardial infarction was recorded in 10 of 521 patients (1.9%) in the no clinical inertia group versus 1 of 165 (0.6%) in the clinical inertia group.

### 4.2. Microvascular Complications

Clinical inertia was significantly associated with DN in our study. However, clinical inertia was not associated with a composite of microvascular complications and DR. The findings are congruent with a large randomized controlled trial, ADVANCE study. After a median of 5 years until follow-up, the intensive group reduced the incidence of nephropathy (*p* = 0.006), with no significant effect on retinopathy (*p* = 0.50) [10]. Clinical inertia in our study could decrease HbA1c by 0.08% from baseline. No clinical inertia could reduce HbA1c by 0.35% from baseline. According to this finding, the clinical inertia group decreased HbA1c levels less than the no clinical inertia group. Meeting blood glucose targets is the most successful method to reduce or avoid DN [40]. Hence, clinical inertia, which decreased HbA1c less than the no clinical inertia group, could increase the risk of DN.

A previous study found that lower HDL-C levels are an independent risk factor of microvascular disease, affecting the kidney in type 2 diabetes [41]. The clinical inertia group had HDL-C levels significantly lower than the no clinical inertia group. This possible reason could explain why clinical inertia has been associated with DN.

These findings contrast with the Ostaphan study [23]. The Ostaphan study found that clinical inertia sped up the progression of DR, but not the progression of DN. This outcome could have occurred because the “no clinical inertia group” in our study had significantly higher HbA1c at baseline than those in the “clinical inertia group”. Patients presenting an HbA1c level that is high above the target often experience treatment intensification [24]. They were suspected of having long-term glycemic control issues even before enrollment. The rapid progression of DR could be attributed to the markedly higher mean HbA1c levels [23].

Moreover, the use of insulin was slightly higher in the no clinical inertia group compared with the clinical inertia group. The use of insulin has been identified as a significant predictor of DR onset and progression [42]. This finding could imply that the no clinical inertia group had increased DR levels greater than the clinical inertia group.

Obviously, a lack of universal standard measurement to quantify clinical inertia led to difficulty in comparing the results from related studies [43]. Clinical inertia was described by Ostaphan et al. as a condition in which patients with T2DM had an HbA1c level ≥ 9.0% and had not received treatment intensification with insulin within the previous three months. Our study identified clinical inertia with respect to patients who had HbA1c ≥ 7.0% and did not receive treatment intensification at the index date and subsequent prescription. Our research looked at clinical inertia using a combination of injectable and oral antidiabetic medications (OADs), rather than only injectables or OADs, and evaluated clinical inertia on the index date or the subsequent prescription date.

A post hoc analysis of our study revealed that a decrease in HbA1c by 1% could significantly lower diabetic complications, a composite of microvascular complications, and DN. Similar results were seen in the UKPDS 33 study, the ADVANCE study, the ACCORD study, and the VADT study [7,8,9,10]. Further study is indicated to investigate interventions for enhancing treatment intensification to decrease the incidence of diabetic complications among these patients.

Our findings in this study encountered some limitations. Firstly, this study was a retrospective study in real-world clinical practice, so findings should be considered with caution because of possible confounders and a lack of data. Although we attempted to adjust for potential confounders, potential residual confounding is common in any clinical epidemiological study. Secondly, the data collection of diabetes complications was based on physicians’ entries in medical records during busy patient clinics. The likelihood of incomplete/missing information in these situations is high. Thirdly, patients whose medical cards did not have data on adherence and lifestyle modification were presumed to have good adherence and lifestyle modification. This might not have been true in some cases. This problem is likely to impact the robustness of our results because nonadherence and lifestyle modification affected poor glycemic control and an increased risk of diabetes complications. Finally, the data of those patients admitted with MI, HF, or stroke at other hospitals were unavailable, which may have resulted in an underestimated complication incidence rate.

However, the present study used three propensity score methods to adjust for confounding. The results were in line with the overall cohort study. As a result, the findings from this research have some credibility.

## 5. Conclusions

During 6.48 years of median follow-up, no significance was observed in the effect of clinical inertia on diabetes complications, except for DN, among patients with diabetes receiving overall control of cardiovascular risk factors. These findings require further investigation to understand the associations between clinical inertia and diabetes complications under the same definition of clinical inertia.

## Figures and Tables

**Table 1:** Baseline characteristics comparing between clinical inertia and no clinical inertia groups.

	Overall Cohort				Matched Cohort			
Characteristic	All (*n* = 686)	Clinical Inertia (*n* = 165)	No Clinical Inertia (*n* = 521)	*p*-Value *	All (*n* = 330)	Clinical Inertia (*n* = 165)	No Clinical Inertia (*n* = 165)	*p*-Value *
Sex								
Female	297 (43.3)	83 (50.3)	214 (41.1)	0.039	150 (45.4)	83 (50.3)	67 (40.6)	0.097
Male	389 (56.7)	82 (49.7)	307 (58.9)		180 (54.6)	82 (49.7)	98 (59.4)	
Age (years)	53.59 ± 6.04	53.62 ± 5.97	53.58 ± 6.07	0.931	53.84 ± 6.04	53.62 ± 5.97	54.05 ± 6.11	0.524
Duration of T2DM (years)	5 (3–6)	5 (3–6)	4 (3–6)	0.777	4 (3–6)	5 (3–6)	4 (3–6)	0.392
Health Insurance								
Self-pay	40 (5.8)	12 (7.3)	28 (5.4)	0.590	17 (5.2)	12 (7.3)	5 (3.0)	0.296
Civil Servant Medical Benefit Scheme	311 (45.3)	75 (45.5)	236 (45.3)		155 (47.0)	75 (45.4)	80 (48.5)	
Social Health Insurance	250 (36.4)	55 (33.3)	195 (37.4)		116 (35.2)	55 (33.3)	61 (37.0)	
Universal coverage scheme	85 (12.4)	23 (13.9)	62 (11.9)		42 (12.7)	23 (13.9)	19 (11.5)	
Current drinker	71 (10.4)	18 (10.9)	53 (10.2)	0.771	37 (11.2)	18 (10.9)	19 (11.5)	1.000
Current smoker	31 (4.5)	6 (3.6)	25 (4.8)	0.669	12 (3.6)	6 (3.6)	6 (3.6)	1.000
Hypertension	487 (71.0)	115 (69.7)	372 (71.4)	0.694	236 (71.5)	115 (69.7)	121 (73.3)	0.542
Dyslipidemia	415 (60.5)	106 (64.2)	309 (59.3)	0.274	195 (59.1)	106 (64.2)	89 (53.9)	0.073
Gout	7 (1.0)	1 (0.6)	6 (1.2)	1.000	1 (0.3)	1 (0.6)	0 (0.0)	1.000
Charlson comorbidity index score								
1	539 (78.6)	136 (82.4)	403 (77.4)	0.192	265 (80.3)	136 (82.4)	129 (78.2)	0.406
2	147 (21.4)	29 (17.6)	118 (22.6)		65 (19.7)	29 (17.6)	36 (21.8)	
Diabetic nephropathy	69 (10.1)	13 (7.9)	56 (10.8)	0.372	28 (8.48)	13 (7.9)	15 (9.1)	0.844
Diabetic retinopathy	53 (7.7)	4 (2.4)	49 (9.4)	0.002	17 (5.2)	4 (2.4)	13 (7.9)	0.043
Cardiovascular disease	53 (7.7)	18 (11.0)	35 (6.7)	0.094	30 (9.1)	18 (10.9)	12 (7.3)	0.339
HbA1c at baseline	8.32 ± 1.29	8.03 ± 1.04	8.41 ± 1.35	<0.001	8.04 ± 1.06	8.03 ± 1.04	8.05 ± 1.08	0.892
Lipid profile (mg/dL)								
Total Cholesterol	178.38 ± 50.01	175.62 ± 42.19	179.20 ± 52.15	0.531	176.87 ± 54.90	175.62 ± 42.19	178.04 ± 64.86	0.776
Triglycerides	118 (81–171)	119 (83.5–168)	116 (79–171)	0.996	116 (72–164)	119 (83.5–168)	102 (65–162)	0.221
HDL-C	47.66 ± 14.48	44.56 ± 10.92	48.58 ± 15.28	0.010	47.08 ± 12.99	44.56 ± 10.92	49.45 ± 14.34	0.015
LDL-C	104.20 ± 36.10	106.69 ± 34.96	103.46 ± 36.47	0.483	102.77 ± 35.60	106.69 ± 34.96	99.08 ± 36.01	0.171
Blood pressure (mmHg)								
Systolic	134.78 ± 16.16	135.14 ± 14.03	134.67 ± 16.79	0.722	134.91 ± 15.40	135.14 ± 14.03	134.69 ± 16.70	0.791
Diastolic	78.30 ± 9.98	78.92 ± 9.62	78.11 ± 10.09	0.365	78.53 ± 9.57	78.92 ± 9.62	78.13 ± 9.53	0.458
eGFR (mL/min/1.73 m^2^)	87.73 ± 32.40	92.32 ± 30.38	86.27 ± 32.93	0.106	90.43 ± 32.44	92.32 ± 30.38	88.45 ± 34.50	0.408
BMI (kg/m^2^)	26.86 ± 4.71	27.49 ± 5.02	26.66 ± 4.60	0.073	27.08 ± 4.74	27.49 ± 5.02	26.68 ± 4.42	0.154
Number of drugs used	1.86 ± 0.75	1.95 ± 0.79	1.84 ± 0.73	0.093	1.96 ± 0.79	1.95 ± 0.79	1.97 ± 0.79	0.835
Medications								
Insulin	97 (14.1)	16 (9.7)	81 (15.6)	0.072	32 (9.7)	16 (9.7)	16 (9.7)	1.000
Antiplatelet/Anticoagulant	453 (66.0)	102 (61.8)	351 (67.4)	0.220	216 (65.4)	102 (61.8)	114 (69.1)	0.203
ACEI/ARB	469 (68.4)	113 (68.5)	356 (68.3)	1.000	231 (70.0)	113 (68.5)	118 (71.5)	0.631
BB	147 (21.4)	36 (21.8)	111 (21.3)	0.913	69 (20.9)	36 (21.8)	33 (20.0)	0.787
CCB	287 (41.8)	70 (42.4)	217 (41.6)	0.857	141 (42.7)	70 (42.4)	71 (43.0)	1.000
Diuretic	173 (25.2)	41 (24.8)	132 (25.3)	1.000	76 (23.0)	41 (24.8)	35 (21.2)	0.513
Statin	516 (75.2)	121 (73.3)	395 (75.8)	0.535	242 (73.3)	121 (73.3)	121 (73.3)	1.000
Type of physician								
General practitioners	303 (44.2)	79 (47.9)	224 (43.0)	0.532	151 (45.8)	79 (47.9)	72 (43.6)	0.743
Resident	107 (15.6)	25 (15.2)	82 (15.7)		53 (16.1)	25 (15.2)	28 (17.0)	
Specialists	276 (40.2)	61 (37.0)	215 (41.3)		126 (38.2)	61 (37.0)	65 (39.4)	

Continuous variables are expressed in terms of mean ± standard deviation or median (IQR), as appropriate. Frequencies/percentages are used to characterize categorical variables. * For categorical variables, Fisher’s exact test was used to get the *p*-value for statistical significance. For continuous data with a normal distribution, the independent *t*-test was employed and for skewed data, the Wilcoxon rank-sum test was utilized. Abbreviations: mean ± SD, mean ± standard deviation; median (IQR), median (interquartile range); HbA1c, hemoglobin A1c; HDL-C, high-density lipoprotein cholesterol; LDL-C, low-density lipoprotein cholesterol; eGFR, estimated glomerular filtration rate; BMI, body mass index; ACEI/ARB, angiotensin-converting-enzyme inhibitors/angiotensin II receptor blockers; BB, beta-blockers; CCB, calcium channel blockers.

**Table 2:** Hazard ratios showing the effect of clinical inertia on diabetes complications (*n* = 686).

	Overall Cohort				Matched Cohort			
	Clinical Inertia (*n* = 165)	No Clinical Inertia (*n* = 521)	Hazard Ratio (95% CI)	Adjusted Hazard Ratio * (95% CI)	Clinical Inertia (*n* = 165)	No Clinical Inertia (*n* = 165)	Hazard Ratio (95% CI)	Adjusted Hazard Ratio * (95% CI)
Diabetes complications	67 (40.6)	211 (40.5)	1.01	1.09	67 (40.6)	67 (40.6)	1.03	1.24
			(0.77–1.33)	(0.82–1.45)			(0.73–1.44)	(0.87–1.77)
Composite of macrovascular complications	11 (6.7)	30 (5.8)	1.18	1.24	11 (6.7)	9 (5.4)	1.27	1.33
			(0.59–2.35)	(0.60–2.59)			(0.52–3.06)	(0.52–3.44)
Myocardial infarction	1 (0.6)	10 (1.9)	0.33	0.47	1 (0.6)	1 (0.6)	1.01	-
			(0.04–2.60)	(0.06–3.85)			(0.06–16.22)	
Ischemic stroke	6 (3.6)	12 (2.3)	1.57	1.23	6 (3.6)	5 (3.0)	1.22	1.06
			(0.59–4.20)	(0.43–3.55)			(0.37–4.00)	(0.31–3.68)
Heart Failure	4 (2.4)	16 (3.1)	0.79	1.10	4 (2.4)	4 (2.4)	1.07	1.42
			(0.26–2.37)	(0.35–3.45)			(0.26–4.28)	(0.33–6.06)
Composite of microvascular complications	64 (38.8)	195 (37.4)	1.05	1.16	64 (38.8)	62 (37.6)	1.07	1.34
			(0.79–1.39)	(0.86–1.55)			(0.75–1.51)	(0.93–1.92)
Diabetic nephropathy	35 (21.2)	86 (16.5)	1.36	1.51	35 (21.2)	29 (17.6)	1.32	1.72
			(0.92–2.02)	(1.01–2.27)			(0.81–2.16)	(1.03–2.88)
Diabetic retinopathy	40 (24.2)	142 (27.3)	0.86	0.95	40 (24.2)	40 (24.2)	1.01	1.18
			(0.61–1.23)	(0.66–1.36)			(0.65–1.57)	(0.75–1.87)

* Adjusted for blood pressure, LDL-C, and HbA1c at the time of the last visit, aspirin, ACEIs/ARBs, age, sex, smoking, duration of T2DM, and CCI score. Abbreviations: LDL-C, low-density lipoprotein cholesterol; ACEIs, angiotensin-converting-enzyme inhibitors; ARBs, angiotensin II receptor blockers; HbA1c, hemoglobin A1c.

**Table 3:** The difference in mean HbA1c.

Clinical Inertia (*n* = 165)				No Clinical Inertia (*n* = 521)			
	Mean HbA1c ± SD	Mean Difference (95% CI)	*p*-Value *	Mean HbA1c ± SD	Mean Difference (95% CI)	*p*-Value *	*p*-Value **
Mean HbA1c at baseline	8.03 ± 1.04	−0.080 ((−0.32)–0.16)	0.512	8.41 ± 1.35	−0.35 ((−0.50)–(−0.19))	<0.001	<0.001
Mean HbA1c at the last visit	7.95 ± 1.58			8.06 ± 1.63			0.464

* *p*-value was obtained using a paired *t*-test to compare between mean HbA1c at baseline and the last visit in the same group. ** *p*-value was obtained using an independent *t*-test to compare mean HbA1c between the clinical inertia and no clinical inertia groups.

**Table 4:** Cox proportional hazards model of the association between a decreasing HbA1c and diabetes complications (*n* = 686).

	Hazard Ratio (95% CI)	Adjusted HR * (95% CI)
Diabetes complications	0.94 (0.88–1.00)	0.92 (0.86–0.99)
Composite of macrovascular complications	1.04 (0.87–1.24)	1.01 (0.84–1.21)
Myocardial infarction	0.91 (0.65–1.27)	0.87 (0.63–1.20)
Ischemic stroke	1.08 (0.82–1.42)	1.08 (0.81–1.44)
Heart Failure	1.01 (0.79–1.31)	0.96 (0.75–1.22)
Composite of microvascular complications	0.92 (0.86–0.99)	0.91 (0.84–0.98)
Diabetic nephropathy	0.90 (0.81–0.99)	0.89 (0.80–0.98)
Diabetic retinopathy	0.94 (0.87–1.02)	0.94 (0.86–1.02)

* Adjusted for blood pressure, LDL-C at the time of the last visit, aspirin, ACEIs/ARBs, age, sex, smoking, duration of T2DM, and CCI score.

## Data Availability

The corresponding author can provide the data used in this study upon request. The data is not available to the public.

## Appendix A

**Table A1:** Hazard ratios showing the effect of clinical inertia on diabetes complications by propensity score covariate adjustment analysis (*n* = 686).

	Clinical Inertia (*n* = 165)	No Clinical Inertia (*n* = 521)	Hazard Ratio (95% CI)	Adjusted Hazard Ratio * (95% CI)
Diabetes complications	67 (40.6)	211 (40.5)	1.01 (0.77–1.33)	1.15 (0.86–1.53)
Composite of macrovascular complications	11 (6.7)	30 (5.8)	1.18 (0.59–2.35)	1.39 (0.66–2.91)
Myocardial infarction	1 (0.6)	10 (1.9)	0.33 (0.04–2.60)	0.50 (0.06–4.18)
Ischemic stroke	6 (3.6)	12 (2.3)	1.57 (0.59–4.20)	1.40 (0.48–4.11)
Heart Failure	4 (2.4)	16 (3.1)	0.79 (0.26–2.37)	1.22 (0.38–3.88)
Composite of microvascular complications	64 (38.8)	195 (37.4)	1.05 (0.79–1.39)	1.20 (0.90–1.62)
Diabetic nephropathy	35 (21.2)	86 (16.5)	1.36 (0.92–2.02)	1.55 (1.03–2.33)
Diabetic retinopathy	40 (24.2)	142 (27.3)	0.86 (0.61–1.23)	0.98 (0.68–1.41)

* Adjusted for propensity score, blood pressure, LDL-C, and HbA1c at the time of the last visit, aspirin, ACEIs/ARBs, age, sex, smoking, duration of T2DM and CCI score. Abbreviations: LDL-C, low-density lipoprotein cholesterol; ACEIs, angiotensin-converting-enzyme inhibitors; ARBs, angiotensin II receptor blockers; HbA1c, hemoglobin A1c.

**Table A2:** Balance of patient characteristics after IPTW.

Characteristic	Clinical Inertia (*n* = 165)	No Clinical Inertia (*n* = 521)	Standardized Difference
Sex			
Female	50.7%	41.1%	0.193
Male	49.3%	58.9%	−0.193
Age, mean	53.82	53.55	0.045
Duration of T2DM, mean	5.29	5.31	−0.004
Health Insurance			
Self-pay	7.4%	5.6%	0.075
Civil Servant Medical Benefit Scheme	44.2%	45.5%	−0.026
Social Health Insurance	32.9%	37.3%	−0.093
Universal coverage scheme	15.5%	11.7%	0.112
Current drinker	10.0%	10.2%	−0.005
Current smoker	3.6%	4.8%	−0.062
Hypertension	70%	70%	−0.061
Dyslipidemia	60%	60%	0.075
Gout	0.6%	1.1%	−0.062
Charlson comorbidity index			
1	79.8%	77.9%	0.047
2	20.2%	22.1%	−0.047
Diabetic nephropathy	8.4%	10.3%	−0.066
Diabetic retinopathy	3.0%	9.0%	−0.255
Cardiovascular disease	12.5%	6.6%	0.199
HbA1c at baseline, mean	8.25	8.34	−0.072
Lipid profile (mg/dL)			
Total Cholesterol, mean	173.71	178.76	−0.107
Triglyceride, mean	127.43	139.45	−0.119
HDL-C, mean	44.36	48.60	−0.319
LDL-C, mean	105.52	103.19	0.065
Blood pressure (mmHg)			
Systolic, mean	135.13	134.65	0.031
Diastolic, mean	78.96	78.09	0.089
eGFR (mL/min/1.73 m^2^), mean	91.46	86.88	0.144
BMI (kg/m^2^), mean	27.57	26.66	0.191
Number of drug use, mean	1.86	1.86	−0.009
Medications			
The use of insulin	15.3%	14.2%	0.033
Antiplatelet/Anticoagulant drugs	60%	70%	−0.155
ACEI/ARB	70%	70%	−0.016
BB	22.7%	21.2%	0.034
CCB	42.1%	41.7%	0.009
Diuretic	26.4%	25.4%	0.024
Statin	73.0%	76.0%	−0.069
Type of physician			
General practitioners	42.6%	44.0%	−0.029
Resident	16.0%	15.8%	0.008
Specialists	41.4%	40.2%	0.023

Abbreviations: HbA1c, hemoglobin A1c; HDL-C, high-density lipoprotein cholesterol; LDL-C, low-density lipoprotein cholesterol; eGFR, estimated glomerular filtration rate; BMI, body mass index; ACEI/ARB, angiotensin-converting-enzyme inhibitors/angiotensin II receptor blockers; BB, beta blockers; CCB, calcium channel blockers.

**Table A3:** Hazard ratios showing the effect of clinical inertia on diabetes complications by IPTW analysis (*n* = 686).

	Clinical Inertia (*n* = 165)	No Clinical Inertia (*n* = 521)	Hazard Ratio (95% CI)	Adjusted Hazard Ratio * (95% CI)	Double Robustness **
Diabetes complications	67 (40.6)	211 (40.5)	1.14	1.21	1.20
			(0.86–1.50)	(0.91–1.60)	(0.90–1.59)
Composite of macrovascular complications	11 (6.7)	30 (5.8)	1.62	1.49	1.38
			(0.79–3.32)	(0.72–3.09)	(0.62–3.07)
Myocardial infarction	1 (0.6)	10 (1.9)	0.26	0.23	0.23
			(0.03–2.02)	(0.02–2.62)	(0.02–3.09)
Ischemic stroke	6 (3.6)	12 (2.3)	2.28	1.46	2.12
			(0.81–6.38)	(0.60–3.54)	(0.51–8.83)
Heart Failure	4 (2.4)	16 (3.1)	1.14	1.19	1.66
			(0.37–3.50)	(0.47–3.01)	(0.56–4.90)
Composite of microvascular complications	64 (38.8)	195 (37.4)	1.17	1.27	1.25
			(0.87–1.55)	(0.95–1.70)	(0.94–1.67)
Diabetic nephropathy	35 (21.2)	86 (16.5)	1.45	1.72	1.71
			(0.96–2.19)	(1.11–2.67)	(1.09–2.66)
Diabetic retinopathy	40 (24.2)	142 (27.3)	0.96	0.98	1.00
			(0.67–1.37)	(0.68–1.39)	(0.70–1.43)

* Adjusted for blood pressure, LDL-C, and HbA1c at the time of the last visit, aspirin, ACEIs/ARBs, age, sex, smoking, duration of T2DM and CCI score. ** Adjusted for blood pressure, LDL-C, and HbA1c at the time of the last visit, aspirin, ACEIs/ARBs, age, sex, smoking, duration of T2DM, CCI score, insurance, diabetic retinopathy, cardiovascular disease and antiplatelet/anticoagulant. Abbreviations: LDL-C, low-density lipoprotein cholesterol; ACEIs, angiotensin-converting-enzyme inhibitors; ARBs, angiotensin II receptor blockers; HbA1c, hemoglobin A1c.
